# Development of a Configurable Growth Chamber with a Computer Vision System to Study Circadian Rhythm in Plants

**DOI:** 10.3390/s121115356

**Published:** 2012-11-09

**Authors:** Pedro J. Navarro, Carlos Fernández, Julia Weiss, Marcos Egea-Cortines

**Affiliations:** 1 DSIE, Universidad Politécnica de Cartagena, Campus Muralla del Mar, s/n. Cartagena 30202, Spain; E-Mails: pedroj.navarro@upct.es (P.J.N.); carlos.fernandez@upct.es (C.F.); 2 Genética, Instituto de Biotecnología Vegetal, Universidad Politécnica de Cartagena, Cartagena 30202, Spain; E-Mail: Julia.weiss@upct.es

**Keywords:** circadian clock, floral size, diel growth, *Petunia opuntia*, *Antirrhinum majus*, computer vision

## Abstract

Plant development is the result of an endogenous morphogenetic program that integrates environmental signals. The so-called circadian clock is a set of genes that integrates environmental inputs into an internal pacing system that gates growth and other outputs. Study of circadian growth responses requires high sampling rates to detect changes in growth and avoid aliasing. We have developed a flexible configurable growth chamber comprising a computer vision system that allows sampling rates ranging between one image per 30 s to hours/days. The vision system has a controlled illumination system, which allows the user to set up different configurations. The illumination system used emits a combination of wavelengths ensuring the optimal growth of species under analysis. In order to obtain high contrast of captured images, the capture system is composed of two CCD cameras, for day and night periods. Depending on the sample type, a flexible image processing software calculates different parameters based on geometric calculations. As a proof of concept we tested the system in three different plant tissues, growth of petunia- and snapdragon (*Antirrhinum majus*) flowers and of cladodes from the cactus *Opuntia ficus-indica*. We found that petunia flowers grow at a steady pace and display a strong growth increase in the early morning, whereas *Opuntia* cladode growth turned out not to follow a circadian growth pattern under the growth conditions imposed. Furthermore we were able to identify a decoupling of increase in area and length indicating that two independent growth processes are responsible for the final size and shape of the cladode.

## 1. Introduction

The rotation of the Earth imposes a rhythmic change in two physical parameters, light and temperature, that play an important role on plant morphogenesis. The current hypothesis supported by work performed in cyanobacteria, fungi, plants and animals is that an endogenous mechanism has evolved that keeps the time allowing anticipation of processes according to a foreseeable future [1]. The synchronization of cellular and developmental processes with the daytime changes occurs as a result of a coordinated set of genes known as the circadian clock [2]. Leaf movement, volatile emission, growth, photosynthesis capacity or starch levels are just a few of the processes that are regulated by the endogenous rhythmic outputs of the clock [3]. Proper clock function has been linked to increased photosynthesis, growth and fitness in plants indicating that it is a central layer of control [4].

Substantial efforts have been devoted to elucidate the functions and different interactions between the genes that comprise the core clock. Important advances were made with the usage of *Luciferase* reporter gene driven by circadian regulated promoters [5]. These biological models rely on artificial vision systems that record promoter activity as the luciferase protein degrades the luciferin into oxyluciferin. However identification of changes in circadian regulation or circadian regulated processes does not always require a transgenic system, nor is always feasible.

Plant growth has been analyzed using artificial vision setups that measure alternative parameters. Amongst these parameters are growth analysis [6,7] and chlorophyll fluorescent imaging [8–10], used to study oxidative stress and pathogen attack. Lately delayed fluorescence imaging has been used to study circadian regulation in plants [11], but this system can be applied only to leaves or other organs with a strong chlorophyll production.

Computer vision techniques for measuring plant growth are attractive because of the flexibility they offer concerning different setups for image acquisition of the element under study. Indeed approaches using between one and three cameras to obtain data have been described. Growth of corn seedlings has been monitored using a sequence of 506 images from two views, one front and one orthogonal to measure small variations in growth using optical flow [12]. This technique has been used to visualize the growth of root and stem of a plant [13]. Furthermore, two cameras located orthogonally in front of the stem allowed creating a virtual model of the plant that can be seen from any point, reconstructing the images on a computer and thereby obtaining parameters of interest. A different approach used a camera and a near infrared light source (NIR), capturing images every two minutes and getting 50 to 150 images to measure the growth of stem/leaves of corn seedlings at different temperature and lighting conditions, using optical flow calculations [14]. Finally a third approach has been developed to analyze seedling growth and leaf area by taking three pictures from the same plant at every 120° angle interval [15]. To investigate morphological characteristics of different species at the same time, Shimizu *et al.* used a camera mounted on a stepping motor. A computer captured and stored images of 10 *Chrysanthemun* plants placed each 18° apart forming a semicircle [16]. Machine vision system measured the stem elongation during three periods of night/day. The developed system uses a reflective marker to ease the detection of stem growth.

Plant growth follows a rhythmic pattern that is apparently influenced by the photosynthetic physiology *i.e.*, C_3_-based photosynthesis and Crassulacean Acid Metabolism (CAM) plants display different patterns [17], and monocots and dicots also show differences in the way continuous light and temperature affects diel leaf growth [18]. Most leaves and stems of dicotyledonous plants grow during the so-called subjective night [19,20], but knowledge in the development of other organs like flowers is basically missing. Furthermore the rhythmic movement of plant organs known as nutation makes it challenging to obtain quality data to measure and quantify both nutation and growth.

Computer vision techniques for the study of circadian rhythms in plants present a high complexity of implementation primarily due to four factors: (1) need for a comprehensive lighting to control day/night rhythm that will not disrupt the photoperiod, (2) need to consider the nutation, (3) need to consider the phototropism and (4) high computational requirements. In this study we describe a novel approach based on a computer vision system to solve the above challenges.

## 2. System for Circadian Rhythm Assessment

### 2.1. A Flexible Growth Chamber

We have developed a growth chamber to study growth in a 24 h continuous fashion. It is constructed of removable panels of aluminium integrating an artificial vision system. The corresponding vision system comprises an image capture device, an illumination system and an image-processing unit. We designed the growth chamber with a reconfigurable structure to accommodate plants of different sizes. It has an inner volume of 300 × 300 × 700 mm, and an external size of 800 × 900 × 700 mm.

**Figure 1.**
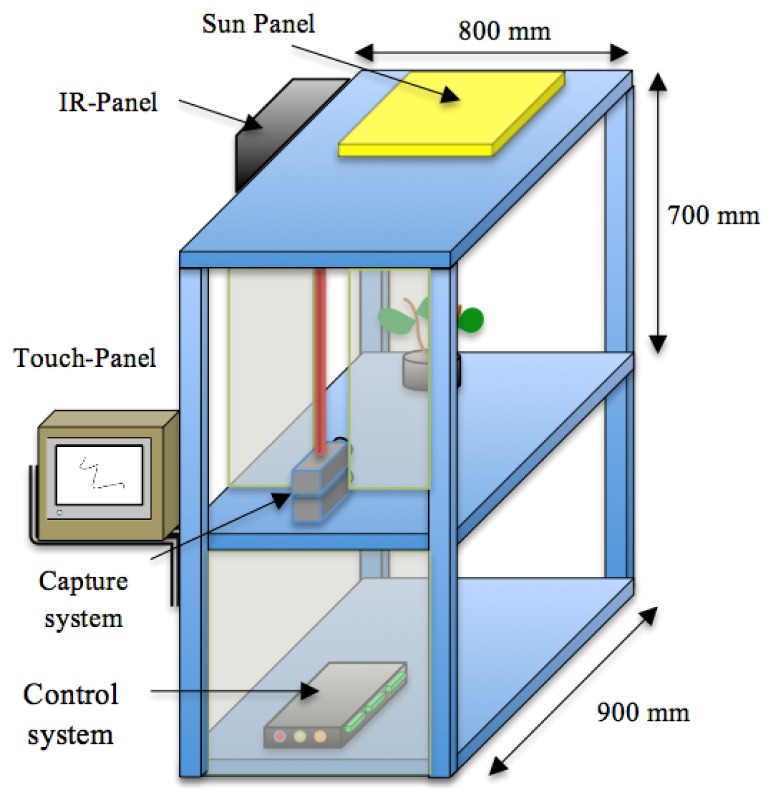
Growth chamber with computer vision system.

The aluminium profiles used for the construction allow the reorganization of the light and cameras, allowing a high versatility to perform experiments with plants of different sizes. Figure 1 shows a schematic representation of the chamber with all the systems that integrate the prototype.

### 2.2. Computer Vision System

The study of circadian events requires an unusual capacity to fetch variations of form and/or shape at time intervals that can be as low as minutes. In practical terms, both nutation and growth have to be separated from each other in order to quantify both parameters. We designed acomputer vision system to analyze the behaviour of circadian rhythm of one or more plants in a continuous way. In order to achieve that, we used two cameras and a configurable software tool, which allows measuring the morphological characteristics during the programmed periods of day/night. The vision system is divided in three main elements as described below.

#### 2.2.1. Image Capture System

**Figure 2.**
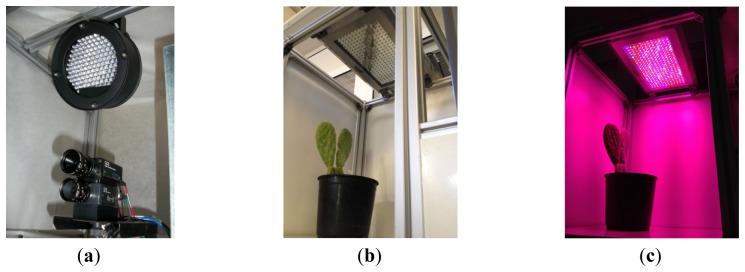
(**a**) CCD cameras and night panel; (**b**) day light panel OFF; (**c**) day light panel ON (the combination of wavelengths makes it look purple to the human eye).

The image capture device comprises two low cost CCD cameras (SONY XC-75CE) mounted on two parallel planes separated by a distance of 3.5 mm and with their optical centers on the vertical axis (Figure 2(a)). One is set up to capture day signals and the second for the night periodto obtain high quality images with the highest sharpness. In both cases the resolution used was of 768 × 576 pixels on a gray scale (8 bit). The interface between the cameras and the image capture device was implemented via a frame-grabber (Matrox Meteor II) with the standard CCIR. Both cameras were equipped with the same lenses (C-mount lens, focal length: 12 mm F1.4) to avoid disparity on the images captured during day and night. A linear polarizing filter was attached to the front of the lens of the “day-camera” to prevent specular reflectance from intense illumination due to the solar-light-panel. The nocturnal camera or darkness-camera did not have a filter in order to increase the sensibility to the far-red spectrum.

#### 2.2.2. Illumination System

**Figure 3.**
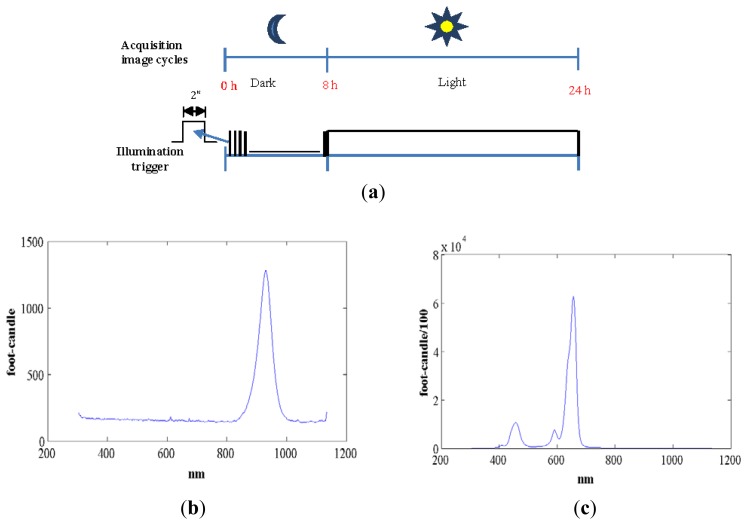
(**a**) Cycling of the illumination system for a circadian period of 8 h dark and 16 h light; (**b**) light spectra for night lighting; and (**c**) light spectra for day lighting.

We used standard agriculture illumination designed for growth improvement. It comprises two panels based on Light-Emitting Diode (LED) technology that provide light at different wavelengths according to a day/night period scheme that is reproduced inside the chamber. The panel emitting visible light is made up of an array of 48 LEDs, combining different wavelengths (see Figure 3(c)). This reproduces the wavelengths necessary to activate photosystem I and II ensuring a proper vegetative and reproductive growth of the plants. Indeed there are at least four types of photoreceptors that play a role in light signalling as inputs during plant development [21]. These include UV-light receptors, red-light, far-red light and at least two additional that have not been characterized in terms of absorption wavelength. The night panel is formed by an array of LEDs with a wavelength of 948 nm (see Figure 3(b)). The far-red light wavelengths were switched on during the dark signalling capture for a few seconds. Nevertheless, far-red light does not interfere with the subjective night and is extensively used in night imaging of photoperiod sensitive experiments [22]. Figure 3(a,b) show light spectra (wavelength *vs.* light intensity). IR peaks for day and night lighting systems do not match because during the night period spectrum must be far-red to avoid any interference in plant's growth.Figure 3(a) shows how the light system is activated and deactivated in the day/night periods of 16 h/8 h. During the night period, the control system activates the night panel for a period of 2 s, enough time for the night-camera to integrate the image without interfering with plant performance or morphogenesis. During the day period, the day panel remains active providing the radiation to allow proper development.

**Figure 4.**
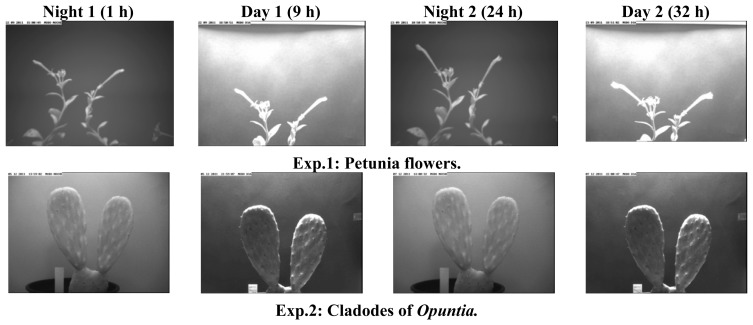
Images taken during two experiments under different illumination conditions.

Figure 4 shows images of two species used to set up the system, petunia flowers and cladodes of the prickly pear cactus *Opuntia ficus-indica*. The first experiment was performed with petunia flowers for a period of 3 days whereas the second experiment was performed using cladodes of *Opuntia* for a period of 15 days.

#### 2.2.3. Image Processing Unit

The image processing unit was developed in C# using the Matrox Imaging Library 9.0 programming libraries [23]. The processing system has two well-defined software modules: (a) an image capture control module and (b) an image analysis module. We describe the functionality of each of the modules:

##### a. Image Capture Module

###### Capture Parameters' Set-up

The image capture control module is in charge of configuring the image capture system and the lighting system. The main functions of this module were:to establish experimental time-frames, to set the initial t*_i_* and final date t*_f_* of the experiment, to establish the length of light periods of circadian rhythm (hours of day and night e.g., 16 h/8 h), to set the number of images per hour to be captured (e.g., 1 image/hour), to target the experiment and select the image format (BMP/TIFF/JPG/PNG) for processing, to set the time of switching on/off the night panel during the night period (e.g., 2 s), to set the timing of on/off pre-switching of the illumination panels. This time is necessary to have the light panels at full performance before the signal capture is made and to set distance of calibration between the plant and the cameras.

###### Camera Calibration

**Figure 5.**
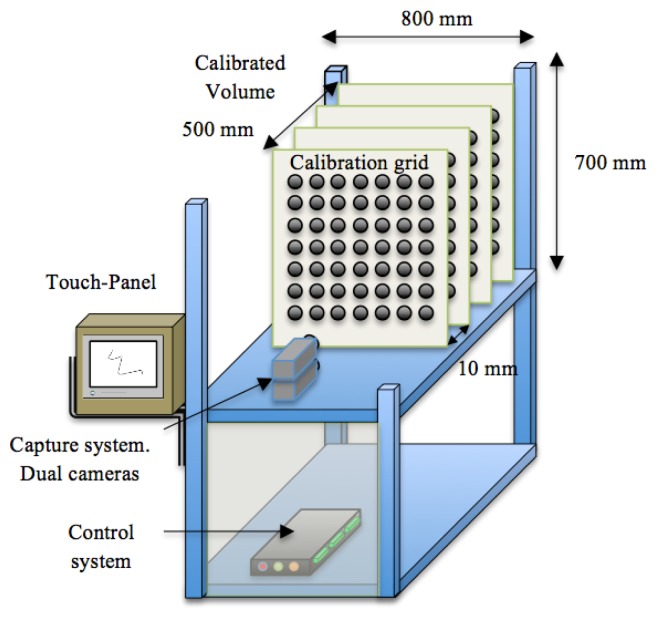
Calibration planes used to obtain real measurements from the growth chamber.

In order to calibrate the capture system [24] we used a grid of 100 circles distributed in ten columns and ten rows. Each circle had a radius of 5 mm, and separation between circles was 10 mm. The volume of the growth chamber used for image capture was divided into 50 planes separated 10 mm from each other and perpendicular to the plane of the camera (Figure 5). Each camera was calibrated for each of the planes. The calibration information, together with the distance between the cameras and the plane of calibration are stored in the image processing system. Before starting the image capture process, the image capture module requests to input by the user the distance of the sample in order to calibrate the system.

The number of images generated for a single experiment depends on the images per hour and the time span of the measurements (see Table 1.).

As the number of images requiring processing is very high, we programmed an automated image analysis module.

##### b. Image Analysis Module

Once image capture ends, the image analysis module is in charge of extracting the relevant information of the experiment. The image analysis module allows the user to process the captured images to analyse growth and movement from all aerial organs (stems, leaves and flowers). We developed a software tool that allows the automatic measurement of the morphological characteristics like area, length, angles, perimeter and centre of gravity. It also allows obtaining indirect parameters as compactness, motion vector and growth speed on a set of images determined by the user.

**Figure 6.**
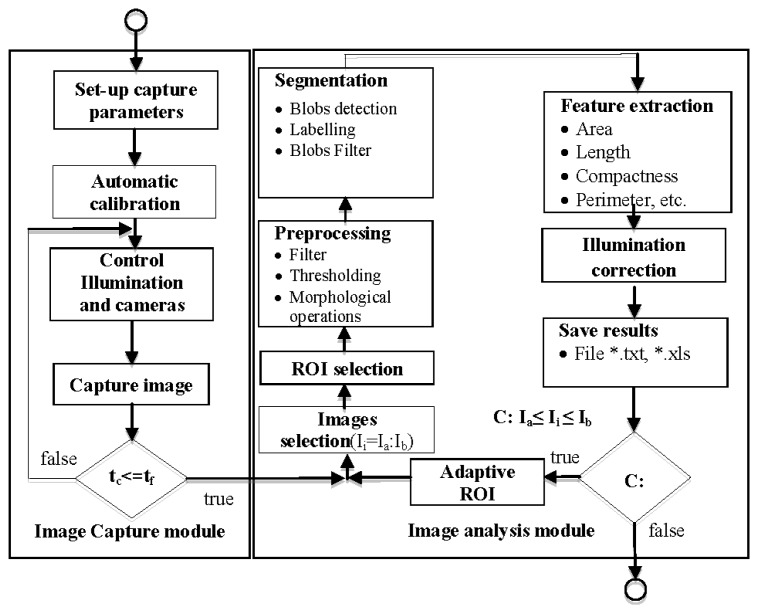
Flowchart of the image-processing unit to analyse the circadian rhythm (being t*_c_* current time, t_f_ final time and I_i_ current image).

The module allows the application of a reconfigurable set of image processing algorithms in an iterative fashion over a set of images corresponding to one experiment. The results obtained are exported by the software module as *.xls or *.txt for representation and interpretation. Figure 6 shows a flowchart of image capture and image analysis modules.

In order to increase the flexibility of the image analysis module, and use it in different experimental set-ups, the user can configure the preprocessing, segmentation and feature extraction. Preprocessing can be configured by the user using different (up to four) filtering (average, median, Laplacian, sharpen, custom mask: 3 × 3, 5 × 5 or 7 × 7) or morphologic (dilation, erosion, opening and closing) operations [25]. Edge detectors (Robert border operator, Sobel border operator, *etc.*) are used in those circumstances where plants organs show kinky aspect. Segmentation can be configured by the user using several algorithms including thresholding (Otsu [26], Kapur [27] or Kittler [28]), blobs detection and blobs filtering by minimal and maximal values of area, compactness, length and perimeter. These can be applied in a sequential manner and in the order decided by the user.The feature extraction stage can also be configured to determine characteristics of segmented organs like number, labelling, area, length, centre of gravity, compactness, perimeter, angle of the principal axis, and register these values.

**Figure 7.**
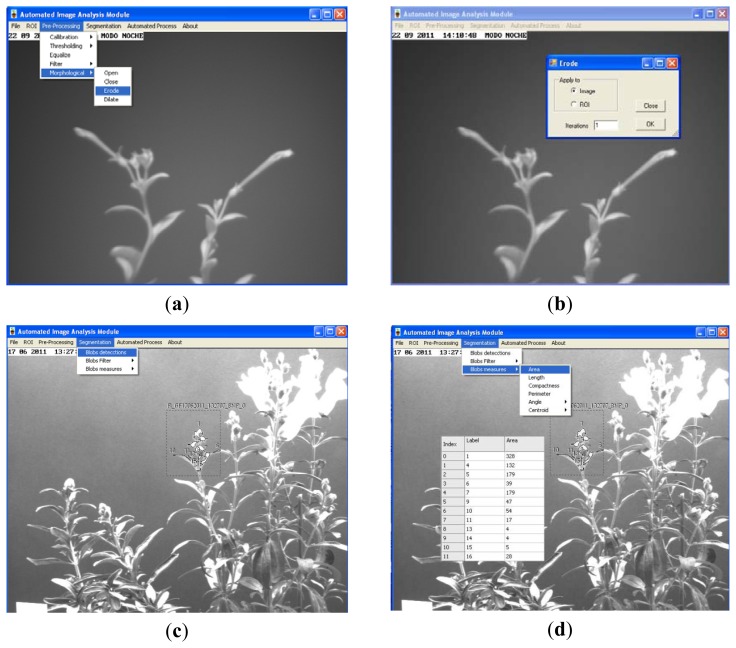
(**a**) Selection of morphological operators; (**b**) Configuration of erosion; (**c**) Blob detection in a ROI; (**d**) Compute area over selected blobs.

The full algorithm is applied to the n images—I_a_ to I_b_—comprising every analysis (see Figure 6). Figure 7(a,b) shows an example of the configuration process for the morphological operators; configuration comprises parameters such as the number of iterations. Figure 7(c) shows blob calculation over a ROI and Figure 7(d) shows the area calculated from the blobs in Figure 7(c).

**Figure 8.**
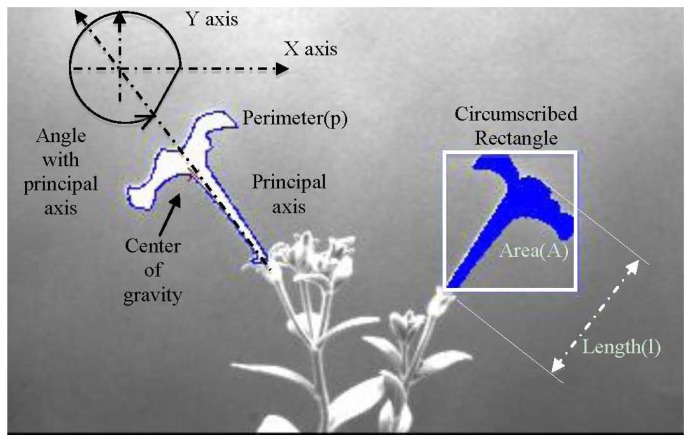
Calculation of features -angle, center of gravity, perimeter, area and length- from *Petunia* flowers.

Figure 8 shows two petunia plants with several flowers at different developmental stages. We depict some of the characteristics computed on a petunia flower at late stages of development.

Starting from morphological parameters like area, length and center of gravity (GC), we calculated compactness (C), motion vector (MV) and growth velocity (GV).

The following equation is used to calculate the compactness:

(1) $$C=\frac{p^{2}}{4\pi A}$$

where *A* and p are equal to the area and the perimeter of the flower. Compactness values of 1 indicate a circular shape.

MV represents the displacement module of the centroids (*cX*_i_, *cY_i_*) of an organ—*i*—referred to its origin (*cX_o_*, *cY_o_*). Origin means the position of the centroids in the first image of the experiment:

(2) $$\text{MV}=\sqrt{{\left(cX_{i}-cX_{o}\right)}^{2}+{\left(cY_{i}-cY_{o}\right)}^{2}}$$

We can interpret the motion vector as a magnitude reflecting the organ activity *i.e.*, positive and negative values in a short temporal range indicate high mobility for an organ. In contrast, small changes in slopes indicate low movement activity.

The growth velocity at point *j* was computed according to:

(3) $$v_{j}=\frac{dl_{j}}{dt}$$

being *l_j_* the distance between two measures *j* and *j* + *1*.

### 2.3. Plant Material

We used three types of plants: the ornamentals *Petunia x hybrida* and *Antirrhinum majus* line 165E and the cactus *Opuntia ficus-indica*. We grew the double haploid Mitchell line W115 from petunia and *Antirrhinum majus* line 165E using standard greenhouse conditions as described previously [29,30]. Plants grown under natural conditions were transferred to the developed growth chamber that had been set up to respect the day-night rhythm of the year time. Nevertheless, plants were grown inside the chamber in order to achieve perfect synchronization with the artificial day-night cycling for 6 days before sampling started.

*Antirrhinum* plants corresponded to 165E wild type plants and the mutant *nana* [31], identified as a circadian clock mutant in transcriptomic experiments (Egea-Cortines *et al.*, unpublished observations).

Cladodes of the *Opuntia ficus-indica* var. Ofer [32] were planted in pots and grown as described [33] under natural conditions in the greenhouse. Plants were brought to the growth chamber and left for 6 days before sampling started.

In order to select the organs for automatic sampling of morphological characters allowing growth analysis we established three criteria:

1. Organ orientation from the camera. We selected organs that display a lateral profile during the longest period of capture.
2. The position of an organ in relation to other organs. Those organs that are partially or completely overshadowed by a second organ are discarded.
3. Its activity. In the calculation of nutation, we will select those organs that show a higher level of movement and those with a lower movement are discarded.

## 3. Results and Discussion

### 3.1. Adaptive Region of Interest (ROI)

Preliminary experiments performed to set up the system described in this work showed that in many cases plant organs grew out of the range of the Region of Interest (ROI) originally set. Indeed, stems from wild-type and *nana* mutants of snapdragon consistently grew out of the ROI as a result of sheer growth, nutation and in some cases organ deterioration with aging. Thus we had to develop a system that would recalculate the ROI position and size. Plant organs showed different growth and mobility patterns as we could ascertain in the current work. Both parameters changed with differing speed. Furthermore, we could discriminate between slow movements registered for growth and stem circumnutation and fast movements identified in flower growth and opening.

In order to measure a single organ, the Automated Image Analysis module allows selecting manually a ROI where a set of computer vision methods will be applied. Under slow movements, selection of a ROI was in most cases enough to measure. However, during rapid movements like flowering, it was necessary to change the ROI shape.

**Figure 9.**
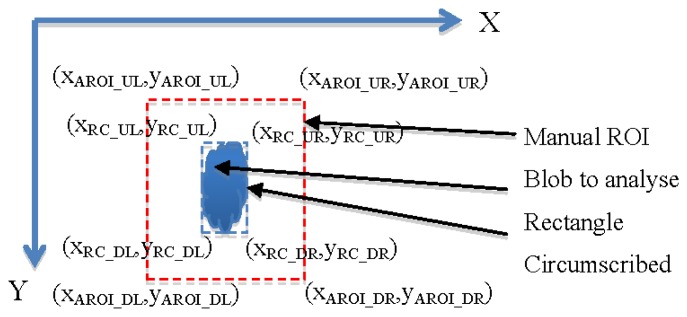
A manually selected ROI and a plant organ circumscribed by a rectangle.

The algorithm to obtain an Adaptive ROI (AROI) was developed in such a way that it calculated the rectangle circumscribing the organ studied, and maintained a constant distance between the rectangle and the ROI. It comprises the following steps:

1. In the first image, the user selects manually a ROI. Within the ROI, a blobcorresponding to the organ studied is determined.
2. For every new image the AROI is calculated. It maintains a constant distance with the vertices of the rectangle containing the blob (see Figure 9). Calculations are done according to expressions on Table 2. The constants MGX and MGY correspond to the maximum growth rate per unit of time in the direction of the X or Y axis and RC being the rectangle circumscribed.

**Figure 10.**
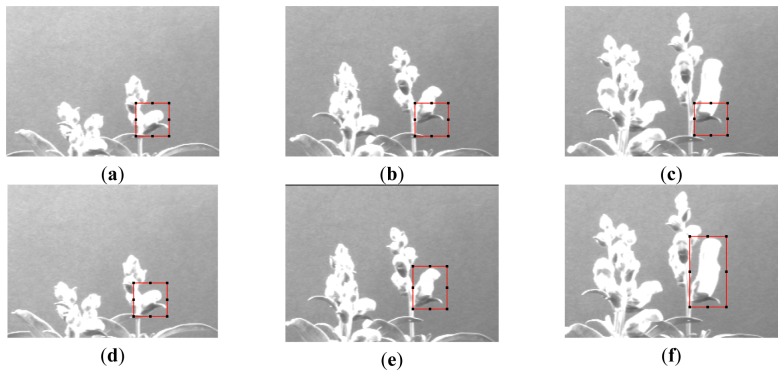
(**a**,**d**) Manual ROI selection; (**b**,**c**) error in ROI selection of the target; (**e**,**f**) Adaptive ROI selection of the target.

Figure 10(a,b,c) show a sequence of three images of *Antirrhinum* flowers taken 24 h apart where the originally fixed ROI is not capable of obtaining the image characteristics as growth has displaced its position. Figure 10(d,e,f) show the same sequence, where the displacement and size increment of the ROI allow the correct acquisition of the object parameters.

### 3.2. Illumination Correction

**Figure 11.**
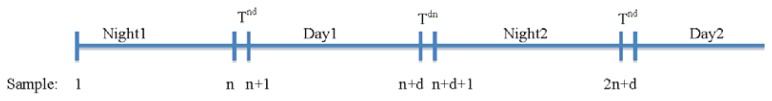
Sequence of night-day (T^nd^) and day-night (T^dn^) transitions.

**Figure 12.**
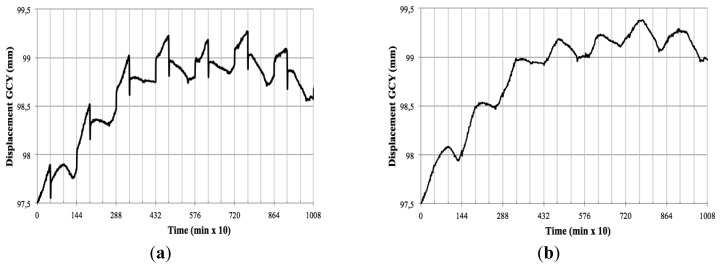
Displacement on the center of gravity center of an *Opuntia* cladode. (**a**) without correcting algorithm; (**b**) after application of correcting algorithm.

Using two independent illumination systems for day and night and two cameras for each period allows a better adjustment than those systems based on a single illumination and camera combination [16]. It allows an exact adjustment of the vision system parameters *i.e.*, optic opening, exposure times and camera adjustments for every period. As a result better images with higher contrast can be obtained in each period, facilitating the segmentation process and follow up of the plant organs. Furthermore, it reduces the complexity of the algorithms used and a higher resolution can be achieved for morphological studies. Figure 11 shows how samples are acquired in a sequence (nigh1-day1-night2-day2 …) generating two types of transitions: night-day (T^nd^) and day-night (T^dn^). This process has as major disadvantage the abrupt transition between grey levels between the day/night shift periods (see Figure 12(a)). We solved this problem by using Equations (4) and (5) to correct the values of variables based on pixel counting like area, length, centre of gravity and perimeter.

Being V*_j_* a variable depending on the pixels counting of the image at instant j, the corrected value of the variable due to a transition from a night period (j = 1, …, n) till a day period (j = n + 1, … n + d) is obtained using Equation (4), while the corrected value of the variable due to a transition from a day period (j = n + 1, …, n + d) till a night period (j = n + d + 1, … 2n + d) is obtained using Equation (5). Being *n* the number of measures along a night period and being *d* the number of measures along a day period:

(4) $$\begin{array}{c} V_{j}^{nd}=V_{j}+K_{1}+K_{2},j=1,\ldots ,n,n+1,\ldots ,n+d\\ K_{1}=V_{n+1}-V_{n}\\ K_{2}=\frac{1}{n-1}\sum_{j=1}^{n-1}V_{j+1}-V_{j} \end{array}$$

(5) $$\begin{array}{c} V_{j}^{dn}=V_{j}+K_{3}+K_{4},j=n+1,\ldots ,n+d,n+d+1,\ldots ,2n+d\\ K_{3}=V_{n+d+1}-V_{n+d}\\ K_{4}=\frac{1}{d-1}\sum_{j=n+d+1}^{2n+d-1}V_{j+1}-V_{j} \end{array}$$

Figure 12(b) shows the results obtained after applying the correction algorithm on the Y displacement of the centre of gravity of an *Opuntia* cladode for a complete experiment comprising 7 days at an acquisition rate of 60 images per hour.

As we can observe, the correction suppresses the sharp changes due to illumination transitions, giving a continuous value of displacement on the gravity centre in the Y axis (GCY).

### 3.3. Petal Growth in Petunia

Studies using*Arabidopsis* plants have shown that hypocotyl elongation occurs during the night period [34], and leaves of *Populus* tobacco and *Arabidopsis* maintain a near-dusk diel growth pattern even in *in vitro* conditions [19,35]. We used the double haploid cultivar of *Petunia x hybrida* Mitchell as it is highly amenable to transformation and has been extensively used to study flower development [36]. The petunia flower in the Mitchell cultivar highly resembles the *P.axillaris* flower as it has a long tube and a large limb. We used two independent plants to monitor growth of several flowers. However, measuring the development of the flower required the decomposition of growth into:

1. the nutation angle of the flower referred to the horizontal axis
2. displacement of the calculated center of gravity of the flower in the X and Y axis
3. floral area
4. floral length
5. Motion vector (MV) calculated from the centre of gravity displacement that would correspond to floral nutation.

**Figure 13.**
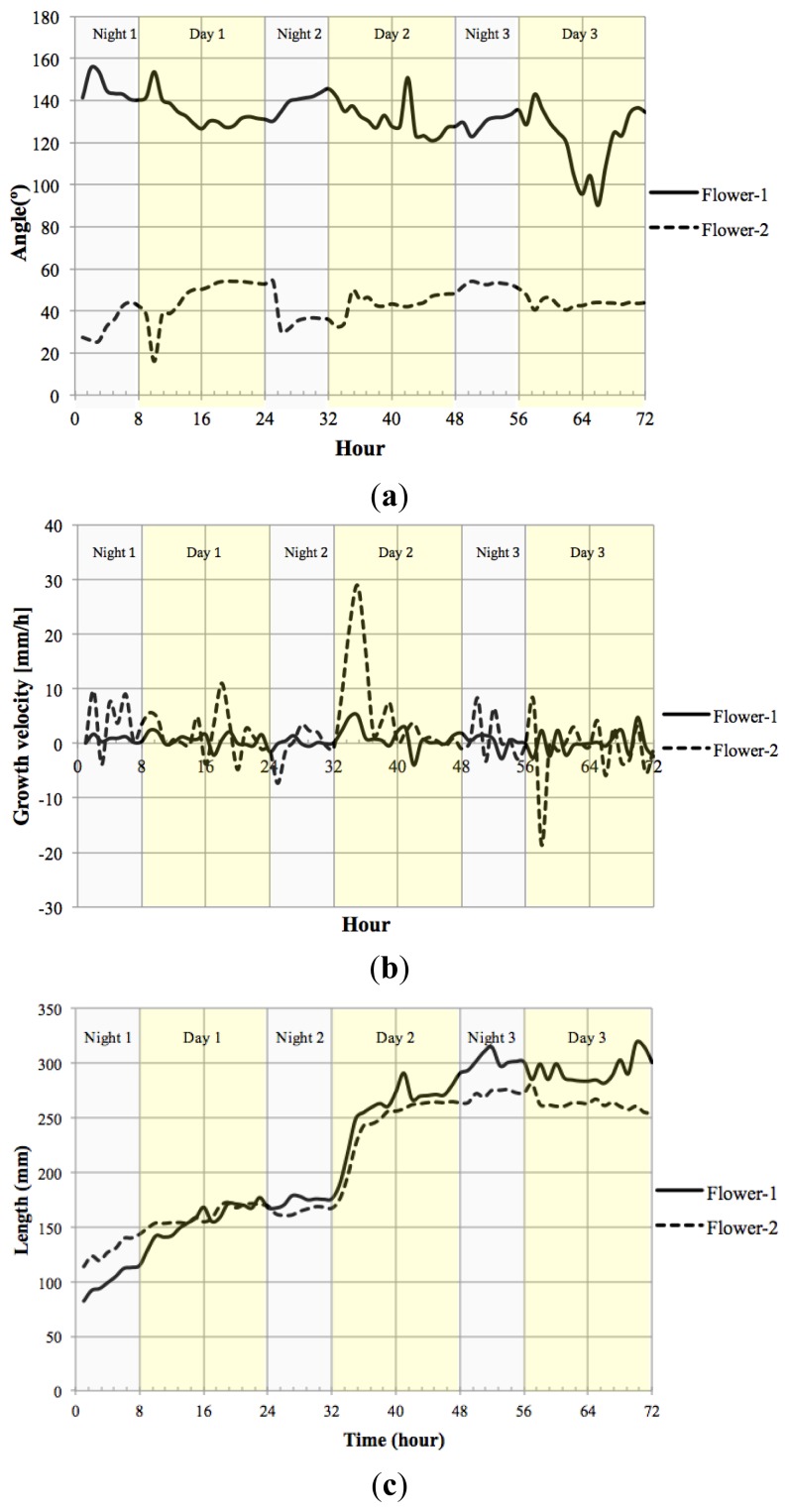

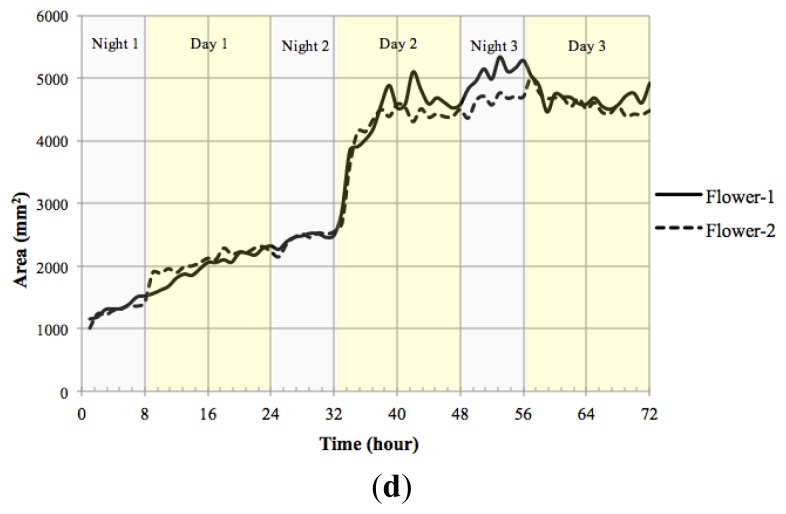
Floral *Petunia*. (**a**) angle; (**b**) growth velocity; (**c**) length; (**d**) area.

We measured the nutation angle of the *Petunia* flowers during development. We observed larger changes in the Y axis as would be expected. Interestingly flowers showed a single pulse of movement shortly after dawn and dusk visible as changes in angle referred to the horizontal axis (Figure 13(a)). Although there were marked differences between flowers, during early stages of development, they tended to maintain a roughly similar angle of circa 50° during the day. At later stages of development, different flowers tended to adopt a final angle that could differ from each other by as much as 40°.

Petal growth is comprised of two periods, an initial period where cell division is responsible of most of the growth and a second period that is due to cell expansion [37]. We observed an almost linear increase in floral length during a period of two days before flower opening. The third day coincided with a sharp increase in floral length that almost doubled its size in a time span of less than 4 h (Figure 13(c)) coinciding with the measurements of floral length, total floral area showed an increase at the beginning of day 3 (TZ2-6; Figure 13(d)).

We calculated the velocity of growth for time zero till time 72 h Figure 13(b). Although total floral length and area do not decrease throughout the stages analyzed, the corresponding relative growth speed was sometimes negative indicating that the flowers had modified their position towards the camera.

**Figure 14.**
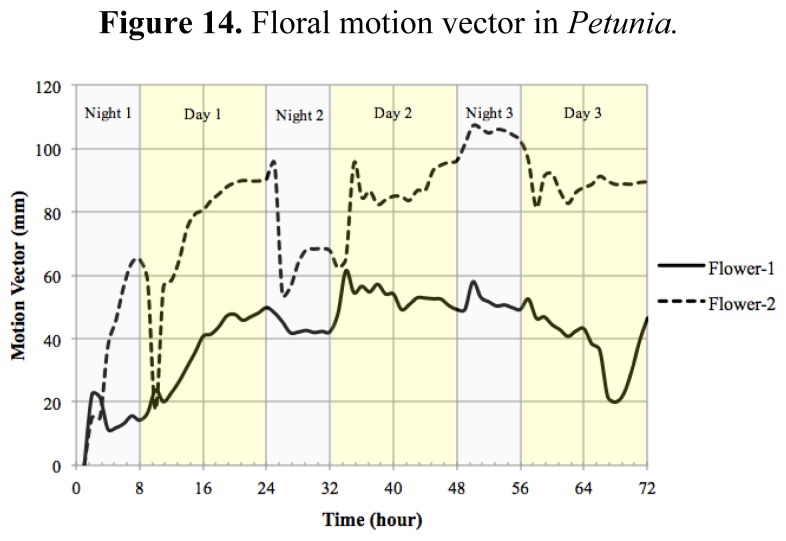
Floral motion vector in *Petunia*.

The motion vector—a measurement of nutation—showed a sharp change shortly after dawn (Figure 14). As flowers matured and the limb expanded to its full size, the motion vector showed a lower activity. We calculated the absolute movement covered by the flowers as a function of age and found that throughout development there was a quick movement of the GC of the flower shortly after dawn (Figure 14). In contrast to floral length and area that showed a single strong increase in size at dawn and early morning, the MV of the flowers presented a progressive dampening, as young flowers had larger changes than mature flowers. All above measures have been obtained with a resolution of one image every 10 min for a 3 days period.

Automatic sampling based on computer vision systems is an important asset as the number of samples taken allow a finer dissection of the process and avoids aliasing, *i.e.*, the imputation of a lack of rhythm or false negative identification of differences as a result of samples being taken too wide apart. The data obtained with the aforementioned computer vision system shows that contrary to the expected growth of leaves that occur during the night, the largest change in petal size occurs in a very short period and happens at dawn and early morning. This is an interesting feature of the *Petunia* petal as scent production in *Petunia* is circadian regulated and happens at night [38]. Our experimental prototype also allows us to develop further tools to study effects of photoperiod on floral development, an important aspect for the ornamental industry.

### 3.4. Cladode Growth in Opuntia

In order to test our system with a plant of a different structure, we analysed growth of cladodes of the prickly pear *Opuntia ficus-indica*. We introduced plants in the growth chamber for a period of 7 days with a photoperiod of 16 h light and 8 h dark. The measurement resolution was of one image every 10 min and the amount of data gathered was 1,008 per variable measured.

**Figure 15.**
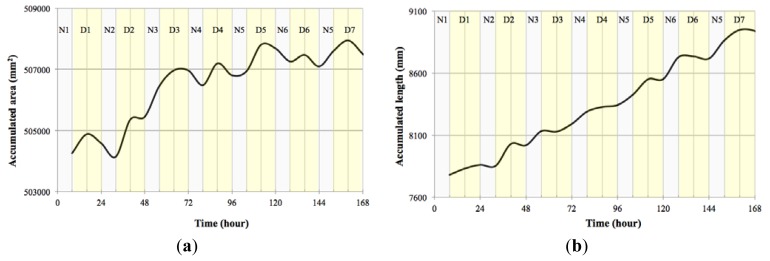
*Opuntia*. (**a**) Accumulated area; (**b**) Accumulated length.

**Figure 16.**
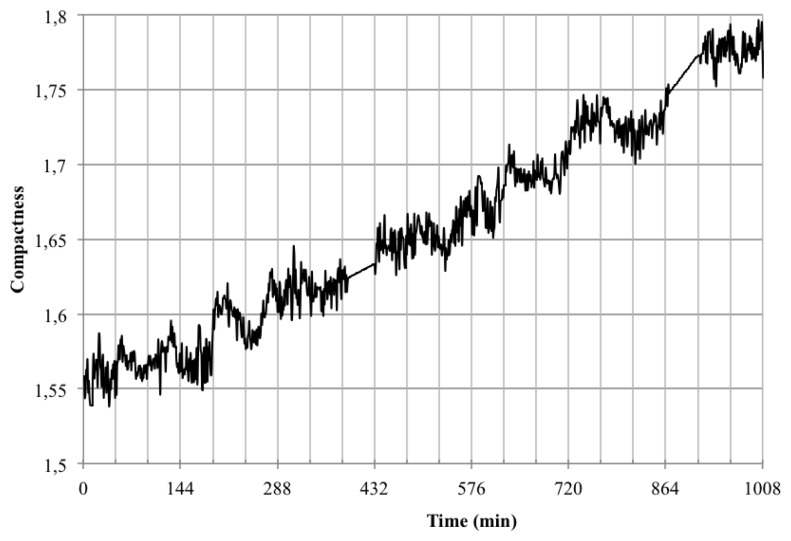
Compactness of *Opuntia*.

Converting the data obtained to accumulated area and length (Figure 15) showed that indeed *Opuntia* cladodes show a rhythmic pattern, however the growth pattern did not adjust to a diel pattern. In fact we uncovered that the accumulated area and length did not directly match as there were certain measured times that registered growth in length but not in area (Table 3). This decoupling of proximo-distal *versus* lateral axis growth coincides with the current genetic model of leaf area as two independent sets of genes controlling these processes. However the cladode is not a true leaf, but a modified stem. Thus the data obtained with the system developed allows the development of testable hypothesis about the genetic and environmental control of growth in cladodes that should share components with the leaves. Looking at the compactness of the cladodes (Figure 16) we also could confirm the previous assumption as during growth, compactness departed from a perfect circle that would be represented by a value of 1.

## 4. Conclusions

Manual data gathering is especially cumbersome when studying biological rhythms as the number of samples increases by an order of magnitude compared to non-cycling processes. Computer vision systems can be of great help, not only to gather data but also to discover patterns in biology that would be otherwise impossible to identify. We have developed a novel system to study circadian growth in plants. In order to achieve this aim we have built a growth chamber that can be reconfigured to accommodate small to middle size plants, shrubs and trees. Two aspects are novel in our system, first the usage of two cameras to obtain day and night samples allows a very high resolution required to study growth in plants that do so at night. This is coupled to a far-red light that does not disrupt the photoperiod. During the day the LED system is good enough to support perfect growth even of desert cacti that require high irradiation.Our software is capable of filtering the nutation of the organs thus allowing the measurement of this parameter, controlled by the clock. The system developed in this work is easy to install by competent engineers and has allowed us to uncover that most of the floral growth in *Petunia* occurs in an extremely short timespan of less than four hours, and contrary to the data gathered in other organs, it occurs during the subjective day.

## Figures and Tables

**Table 1.** The number of images generated for a single experiment.

**Exp.**	**N Days**	**Images/hour**	**Total images to process**
1	7	1	7 × 24 = 164
2	7	6	7 × 6 × 24 = 1,008
3	15	6	15 × 6× 24 = 2,160

**Table 2.** Expressions to calculate AROI.

X_AROI UL_ = X_RC UL_ − MGX	X_AROI DL_ = X_RC DL_ − MGX
Y_AROI UL_ = Y_RC UL_ − MGY	Y_AROI DL_ = Y_RC DL_ + MGY
X_AROI UR_ = X_RC UR_ + MGX	X_AROI DR_ = X_RC DR_ + MGX
Y_AROI UR_ = Y_RC UR_ − MGY	Y_AROI DR_ = Y_RC DR_ + MGY

**Table 3.** Binary pattern of *Opuntia* cladodes for accumulated area and length.

	**Day 1**			**Day 2**			**Day 3**			**Day 4**			**Day 5**			**Day 6**			**Day 7**		
**Area**	0	1	0	0	1	1	1	1	0	0	1	0	1	1	0	0	1	0	1	1	0
**Length**	0	1	1	0	1	0	1	0	1	1	1	1	1	1	1	1	1	0	1	1	0
